# Protocatechuic Acid Attenuates Trabecular Bone Loss in Ovariectomized Mice

**DOI:** 10.1155/2018/7280342

**Published:** 2018-07-29

**Authors:** Seon-A Jang, Hae Seong Song, Jeong Eun Kwon, Hyun Jin Baek, Hyun Jung Koo, Eun-Hwa Sohn, Sung Ryul Lee, Se Chan Kang

**Affiliations:** ^1^Department of Oriental Medicine Biotechnology, College of Life Sciences, Kyung Hee University, Yongin 17104, Republic of Korea; ^2^Department of Medicinal and Industrial Crops, Korea National College of Agriculture and Fisheries, Jeonju 54874, Republic of Korea; ^3^Department of Herbal Medicine Resources, Kangwon National University, Samcheok 25949, Republic of Korea; ^4^Department of Convergence Biomedical Science, Cardiovascular and Metabolic Disease Center, College of Medicine, Inje University, Busan 47392, Republic of Korea

## Abstract

Primary osteoporosis is a disease related to excessive bone resorption due to estrogen insufficiency that occurs postmenopause. Protocatechuic acid (PCA), or 3,4-dihydroxybenzoic acid, is a common compound present in numerous plants. Although numerous biological activities of PCA have been identified, its antiosteoporotic function has not been well established. In this study, the antiosteoporotic activity of PCA supplementation was determined in ovariectomized (OVX) female ICR mice at 12 weeks after OVX. The biomechanical properties of a bone were evaluated by microcomputed tomography. The signaling molecules associated with osteoclast differentiation were determined in bone marrow cells through immunoblot or RT-PCR. Oral supplementation with PCA (20 mg/kg/day) significantly ameliorated the OVX-mediated stimulation of osteoclast activity based on decreases in serum levels of receptor activator of nuclear factor *κ*B ligand (RANKL), osteocalcin, and bone alkaline phosphatase and increase in serum osteoprotegerin (each group, *n* = 6; *p* < 0.05). In addition, the OVX-induced decreases in mRNA expression levels of cathepsin K, calcitonin receptor, nuclear factor of activated T cell cytoplasmic 1 (NFATc1), and tumor necrosis factor (TNF) receptor-associated factor-6 (TRAF6) in bone marrow cells were significantly attenuated (each group, *n* = 6; *p* < 0.05). Finally, the loss of trabecular bone and changes in biomechanical properties of a bone were significantly improved by supplementation with 20 mg/kg PCA (each group, *n* = 6; *p* < 0.05). Collectively, our results show that PCA supplement suppressed trabecular bone loss in OVX mice and therefore might be an effective alternative approach for preventing the progression of postmenopausal osteoporosis.

## 1. Introduction

Protocatechuic acid (PCA) is a 3,4-dihydroxybenzoic acid that occurs in nature and has a similar structure to gallic acid, caffeic acid, and vanillic acid [1]. PCA is a very common compound that is present in numerous plants, including *Rubus coreanus* Miquel, *Astragalus membranaceus* Bunge, cinnamon, star anise, medicinal rosemary, and *Sorghum bicolor* L, and in fruits and products of plant origin. PCA possesses biological activity against diabetes, infection, ageing, and inflammation, as reviewed elsewhere [1–3]. PCA is traditionally considered to be nontoxic and a relatively safe compound for oral administration [1].

Bone is a type of dense connective tissue that is composed of ~80% cortical bone (outer layer, compact bone) and ~20% trabecular bone (inner layer, cancellous bone). Based on porosity and unit microstructure, a bone is basically classified as either cortical, which is a compact and dense form, or trabecular, which is a cancellous or sponge form [4]. A bone undergoes continuous remodeling through resorption of old bone and formation of the same amount of newly formed bone at the same place [5, 6]. Bone mineral density (BMD) has been regarded as a surrogate marker for bone strength and an important factor in bone quality [7]. In addition to BMD, bone volume fraction (BV/TV) and bone microstructure are important factors determining bone strength [8]. Osteoporosis is associated with increased risk of a broken bone due to loss of bone mass and deterioration of bone microarchitecture, which increase bone fragility and susceptibility to hip and spine fractures [9, 10]. Osteoporosis can be classified as either primary or secondary osteoporosis according to cause, which includes age, sex hormones, medical conditions, and diseases [10, 11]. Primary osteoporosis, which is classified as type I (postmenopausal osteoporosis) and is frequently associated with fenestrated trabecular bone resorption, occurs between the ages of 50 and 65 years in postmenopausal women [12]. Estrogen deficiency induces receptor activator of nuclear factor *κ*B ligand (RANKL), the key molecule required for osteoclast differentiation, leading to enhanced osteoclast activation and reduced osteoclast apoptosis [13–15]. In the bone environment, upregulated RANKL, RANK, and osteoprotegerin (OPG) expression and increased RANKL/OPG ratio are important determinants of bone mass in normal and pathological conditions [16]. In addition, quantitative analysis of osteoclast-specific gene markers such as tartrate-resistant acid phosphatase (TRAP), cathepsin K, and calcitonin receptor has been an important and reliable method for identifying osteoclastogenic capability [17–19]. Although hormone replacement therapy (HRT) is effective in the prevention and treatment of postmenopausal osteoporosis, it is associated with a high risk of blood clotting, biliary disease, and breast or endometrium cancer. Therefore, it is important to develop safer compounds with fewer adverse effects than estrogen mimetics [20].

Several studies have shown that PCA has beneficial effects on osteoblast and osteoclast cells *in vitro* [21–23]. In *in vitro* experiments, PCA has an inhibitory effect on osteoclast differentiation [21] and a proliferative effect on osteoblasts [23]. Wu et al. reported that PCA reduced the RANKL-induced TRAP activity and osteoclast-specific gene expression such as TRAP, tumor necrosis factor receptor-associated factor-6 (TRAF6), and cathepsin K in RAW264.7 murine macrophage cells [21]. In addition, the RANKL-mediated signaling pathways including mitogen-activated protein kinases, nuclear factor *κ*B, and cyclooygenase-2 could be attenuated by PCA treatment [21]. Park et al. have shown that PCA inhibits RANKL-induced osteoclast differentiation in mouse bone marrow macrophages and lipopolysaccharide-induced inflammatory bone loss in mice [24]. In contrast, Rivera-Piza et al. suggested in 2017 that PCA might enhance osteogenesis in C3H10T1/2 and 3T3-L1 cells [22]. The antiosteoporotic effect of PCA in the ovariectomized state that leads to estrogen deficiency, one of the etiological factors of postmenopausal osteoporosis, has not been confirmed. In the present study, we investigated the preventive effect of PCA against deterioration of bone structural architecture *in vivo* in an ovariectomized (OVX) mouse model using microcomputed tomography (CT) bone analysis. We also examined the suppressive effects of PCA on levels of biological markers involved in activation of osteoclasts, such as bone alkaline phosphatase (BALP), RANKL, OPG, TRAF6, nuclear factor of activated T cell cytoplasmic 1 (NFATc1), cathepsin K, and calcitonin receptor, in the serum or bone marrow from experimental groups.

## 2. Materials and Methods

### 2.1. Reagents

Protocatechuic acid (PCA), RANKL, and 17*β*-estradiol (E_2_) were purchased from Sigma Chemical Co. (St. Louis, MO). Phosphate-buffered saline (PBS) was purchased from Welgene Inc. (Seoul, Korea). Other chemicals not specified were obtained from Sigma. Enzyme-linked immunosorbent assay (ELISA) kits for RANKL and OPG were purchased form R&D Systems (Minneapolis, MN). ELISA kits for osteocalcin and BALP were obtained from Biomedical Technologies Inc. (Stoughton, MA).

### 2.2. Experimental Animals

Six-week-old female ICR mice were purchased from Korea Laboratory Animal Co. (Daejeon, Korea) and acclimatized for 1 week before the experiments. The mice were housed in controlled environments of temperature (22 ± 2°C) and humidity (53 ± 5°C) under a 12 h light/dark cycle. The mice were provided with sterile standard mouse chow and water ad libitum during the acclimation and experimental periods. All animal experiments were performed strictly according to the Guide for the Humane Use and Care of Laboratory Animals and in accordance with the current ethical regulations for animal care and use at Kyung Hee University (KHUASP (SE)-16-003).

### 2.3. Experimental Model

The mice underwent either bilateral laparotomy (sham, *n* = 6) or bilateral OVX (*n* = 24) under anesthesia with tiletamine/zolazepam (Virbac Korea, Seoul, Korea) and xylazine HCl (Bayer Korea, Kyungkido, Korea) using a ventral approach at 7 weeks of age. The surgical procedure was performed under aseptic conditions following ethical regulations for animal care and use. At 1 week after surgery, the mice were randomly divided into five groups (*n* = 6 per group): (1) sham-operated mice orally administered an equivalent volume of PBS to treatment groups (sham control); (2) OVX mice with daily oral administration of PBS (OVX); (3) OVX mice with daily oral administration of PCA at 10 mg/kg body weight (b.w.); (4) OVX mice with daily oral administration of PCA at 20 mg/kg b.w.; and (5) OVX with intraperitoneal injection (i.p.) of 17*β*-estradiol (E_2_) at 0.1 mg/kg b.w./day three times per week. Body weight was measured weekly, and the PCA or E_2_ dose was adjusted accordingly. All experimental mice received their respective treatment for 12 weeks. There was no treatment-related mortality during the experimental course.

### 2.4. Micro-CT Bone Analysis

To determine structural loss of bone architecture, the proximal and distal parts of the right tibias were scanned by *in vivo* microcomputed tomography (micro-CT, Skyscan1076, Skyscan, Antwerp, Belgium), as previously reported [25]. The scan conditions were set at an aluminum filter of 0.5 mm, X-ray voltage of 50 kV, X-ray current of 200 mA, and exposure time of 360 ms. During each scan, the mice were maintained under anesthesia via inhalation of isoflurane (Hana Pharm, Seoul, Korea). The mice were placed in a chamber filled with 5% carbon dioxide for 5 min, and then the isoflurane was adjusted to 1.5% to keep the mice anesthetized. The data were digitalized with a frame grabber, and the resulting images were transmitted to a computer for analysis using Comprehensive TeX Archive Network (CTAN) topographic reconstruction software. The total volume (TV) indicated the inner area of cortical bone. The trabecular bone volume (BV) indicated the total trabecular bone within the total volume. The bone volume percentage was calculated by dividing the trabecular bone volume by the total volume. Cortical bone parameters assessed were bone volume fraction (BV, mm^3^), mean polar moment of inertia (MMI, mm^4^), cross-section thickness (Cs.Th, mm), and bone mineral density (BMD, g/cm^3^). Trabecular bone parameters assessed were bone volume fraction (BV/TV, %), trabecular thickness (Tb.Th, mm), trabecular separation (Tb.Sp), trabecular number (Tb.N, 1/mm), trabecular bone pattern factor (Tb.Pf, mm^−1^), and specific bone surface (BS/BV, 1/mm). Structure model index (SMI), ranging from 0 to 3, characterizes the type of 3D structure of a bone based on a certain amount of rods and plates [26].

### 2.5. Enzyme-Linked Immunosorbent Assay

At the end of the experiment, all animals were fasted for 6 h, and then blood was collected from the abdominal vena cava under anesthesia with diethyl ether. The blood was allowed to clot for 30 min at room temperature, and sera were obtained after centrifugation at 3000 ×g for 10 min at 4°C. The serum levels of RANKL, osteocalcin, and BALP were determined using commercial ELISA assay kits according to the manufacturer's instructions.

### 2.6. Quantitative Real-Time Polymerase Chain Reaction

Total RNA was extracted from the bone marrow isolated from experimental mice using Trizol reagent according to the manufacturer's protocols. A total of 1 *μ*g of total RNA was reverse transcribed in a 20 *μ*l total volume using oligo (dT) primers with the enzyme and buffer supplied in the PrimeScript® II 1st strand cDNA synthesis kit (Takara, Japan). Quantitative real-time PCR was conducted with an MX3005P (Stratagene, USA) using the primers listed in Table 1. For quantitative real-time PCR, SYBR Premix Ex Taq II (Takara, Japan) was used in a 25 *μ*l reaction mixture containing 2 *μ*l cDNA template, 1 *μ*l forward and reverse primer, 12.5 *μ*l master mix, and 8.5 *μ*l sterile distilled water. The thermal cycling profile consisted of a preincubation step at 95°C for 10 min, followed by 35 cycles at 95°C for 15 sec and 59°C for 1 min. Relative quantitative mRNA levels of cathepsin K, calcitonin receptor, TRAF6, NFATc1, and *β*-actin were determined by the comparative cycle threshold method.

### 2.7. Statistical Analysis

Data are represented as mean ± standard deviation (SD). Group differences were assessed with one-way analysis of variance (ANOVA) followed by a modified *t*-test with Bonferroni correction using SigmaPlot software (Systat Software Inc., San Jose, CA, USA). A statistical probability of *p* < 0.05 was considered significant.

## 3. Results

### 3.1. Effects of PCA on Body, Uterus, and Tissue Weight in OVX Mice

**Figure 1:**
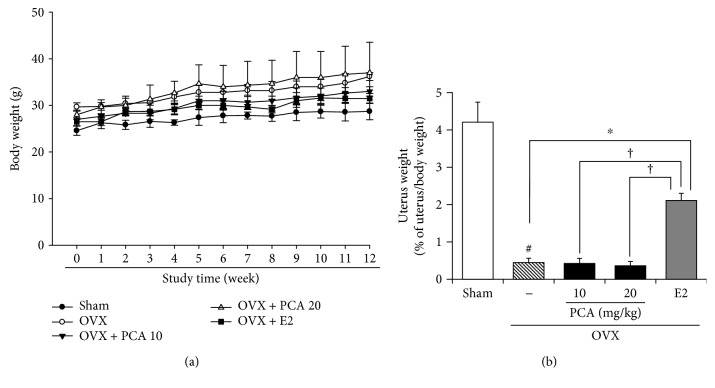
Effects of PCA on changes of body weight and uterus weight in OVX mice. Sham or OVX mice were orally administered vehicle or PCA (10 and 20 mg/kg b.w./day) for 12 weeks. As a positive control group, E_2_ (0.1 mg/kg b.w./day) was administered three times a week for 12 weeks via i.p. injection. Body weight was measured once a week (a). At the end of the experiment, the uterus was removed and weighed (b). Results are expressed as relative ratio per body weight. Data are mean ± SD (each group, *n* = 6). ^#^*p* < 0.05 between the sham and OVX-alone group. ^∗^*p* < 0.05 among OVX groups with or without interventions. ^†^*p* < 0.05 among OVX groups with interventions. b.w.: body weight; i.p.: intraperitoneal; E_2_: 17*β*-estradiol; OVX: ovariectomized; PCA: protocatechuic acid.

There were no clinical signs or abnormalities in behavior during the experiment. The body weight of OVX mice was higher than that of the sham controls (Figure 1(a)), but relative uterus weight per body weight was decreased compared to the sham controls (Figure 1(b)). The uterus is one of the most estrogen-responsive reproductive tissues and therefore can easily become atrophied in the absence of estrogen. E_2_ treatment with 0.1 mg/kg body weight/day administered i.p. significantly increased the relative uterus weight per body weight (*n* = 6, *p* < 0.05). In contrast, PCA supplementation at the dose of 10 and 20 mg/kg body weight/day did not recover the relative uterus weight (Figure 1(b)). The spleen and thymus, major organs of the immune system, are also highly susceptible to estrogen insufficiency. In the OVX group, the weight of the thymus was significantly increased, but that of the spleen was decreased compared to the sham controls (each group *n* = 6, *p* < 0.05; Table 2). Changes in weight of the spleen and thymus were significantly attenuated by E_2_ supplementation. A similar effect was achieved with PCA supplementation at 20 mg/kg (Table 2).

### 3.2. Effect of PCA on the Structural Properties of Cortical Bone

**Figure 2:**
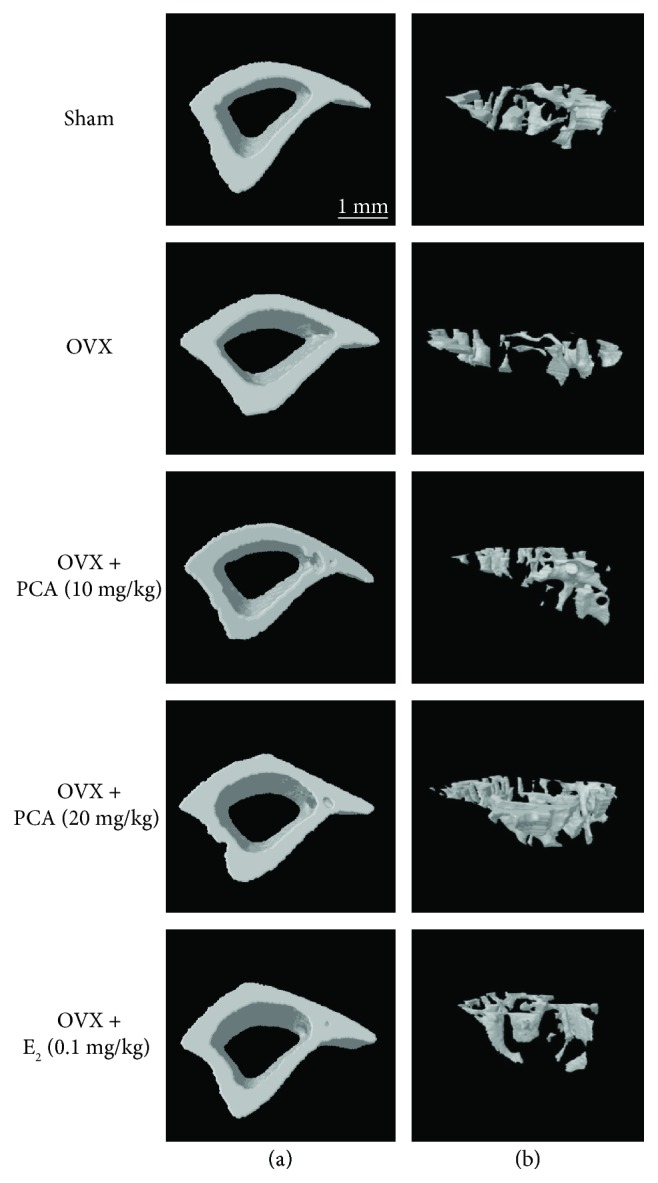
Micro-CT analysis of the cortical and trabecular bone. OVX mice received PBS as a vehicle, PCA (10 mg/kg and 20 mg/kg), or E_2_ (0.1 mg/kg) for 12 weeks. Vehicle and PCA were delivered orally, and E_2_ was given by intraperitoneal injection. The proximal and distal parts of the right tibias were scanned by *in vivo μ*-computed tomography (CT). Representative images of the cortical (a) and trabecular bone (b) reconstructed using Comprehensive TeX Archive Network (CTAN) topographic reconstruction software are presented. E_2_: 17*β*-estradiol; OVX: ovariectomized; PCA: protocatechuic acid.

**Figure 3:**
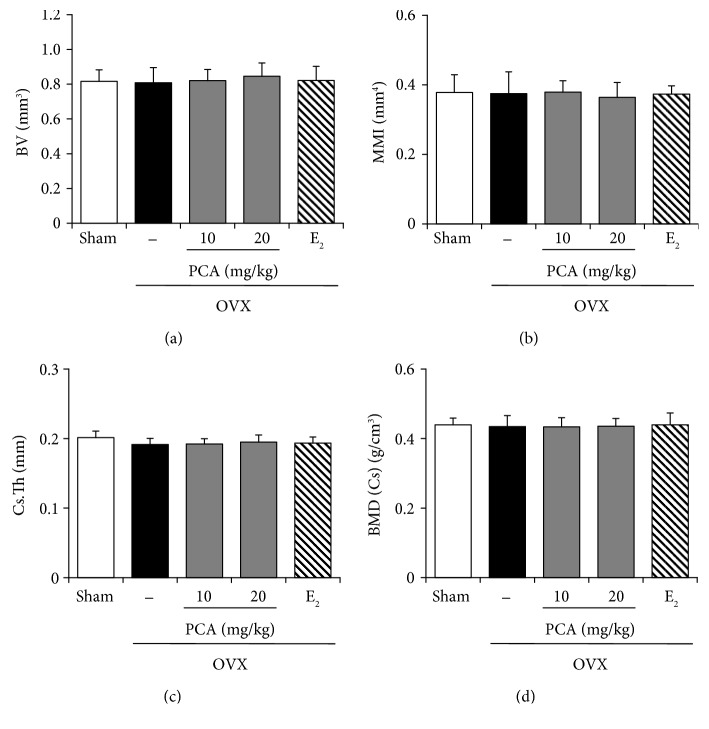
Effect of PCA on the cortical bone in OVX mice. After obtaining the three-dimensional image shown in Figure 3(a), changes in the following parameters of the cortical bone were analyzed: (a) bone volume density (BV), (b) mean polar moment of inertia (MMI), (c) cross-section thickness (Cs.Th), and (d) bone mineral density (BMD). The results are expressed as mean ± SD (each group, *n* = 6). There was no statistical significance among the groups. E_2_: 17*β*-estradiol; OVX: ovariectomized; PCA: protocatechuic acid.

To identify the effect of PCA on structural characteristics of a bone induced by OVX, we analyzed structural parameters for the entire cortical bone of the tibia (BV, MMI, Cs.Th, and BMD) using micro-CT images after scanning the tibia of each mouse (Figures 2(a) and 3). In the OVX group, the values of BV (*n* = 6, *p* > 0.05), MMI (*n* = 6, *p* > 0.05), Cs.Th (*n* = 6, *p* > 0.05), and BMD (*n* = 6, *p* > 0.05) did not change compared to those of the sham controls (Figure 3). In addition, treatment with PCA (10 and 20 mg/kg) or E_2_ did not induce any significant changes in structural parameters of cortical bone of the tibia (Figure 3).

### 3.3. Effects of PCA on the Mechanical Properties of Trabecular Bone

**Figure 4:**
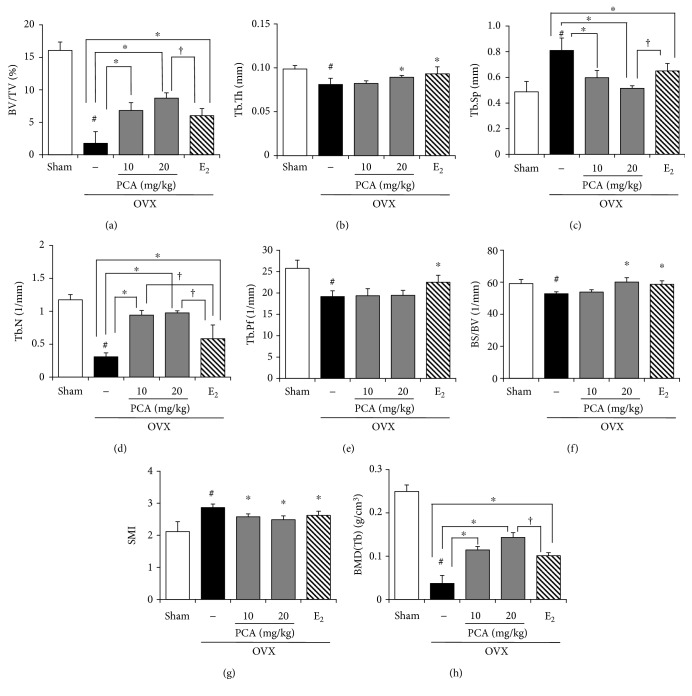
Effect of PCA on the trabecular bone in OVX mice. After obtaining the 3-dimensional image shown in Figure 3(b), changes in the following parameters of the trabecular bone were analyzed: (a) bone volume fraction (BV/TV), (b) trabecular thickness (Tb.Th), (c) trabecular separation (Tb.Sp), (d) trabecular number (Tb.N), (e) trabecular bone pattern factor (Tb.Pf), (f) specific bone surface (BS/BV), (g) structure model index (SMI), and (h) bone mineral density (BMD). The results are expressed as mean ± SD (each group, *n* = 6). ^#^*p* < 0.05 between the sham and OVX-alone group. ^∗^*p* < 0.05 among OVX groups with or without interventions. ^†^*p* < 0.05 among OVX groups with interventions. E_2_: 17*β*-estradiol; OVX: ovariectomized; PCA: protocatechuic acid.

Alteration of trabecular architecture is considered an important component of postmenopausal osteoporosis that influences bone strength [6, 13]. Unlike cortical bone (shown in Figure 3), the trabecular bone structure was significantly affected by OVX. The values of BV/TV, Tb.Th, Tb.N, Tb.Pf, BS/BV, and BMD in trabecular bones were significantly decreased, whereas the values of Tb.Sp and SMI were markedly higher than those of the sham controls (Figures 2(b) and 4). Osteoporotic trabecular bone shows less connectivity and thinner rod-like structures than normal trabecular bone, indicating that the value of SMI is negatively correlated with trabecular bone strength [27]. The trabecular bone of PCA- (10 and 20 mg/kg) treated OVX mice had a more compact trabecular bone structure, with higher bone volume fraction (Tb. BV/TV) and increased trabecular number (Tb.N) and thickness (Tb.Th) with less trabecular separation (Tb.Sp) at both the radius and tibia compared with that of the untreated OVX group (Figure 4) [28]. When compared to the OVX group, BV/TV, Tb.Th, Tb.N, BS/BV, and BMD (Tb) were enhanced, and Tb.Sp and SMI were suppressed in the PCA- (10 and 20 mg/kg) and E_2_-treated groups, whereas Tb.Pf was not significantly different from that in the OVX group (Figure 4(e)). In view of improving BV/TV (Figure 4(a)), Tb.N (Figure 4(d)), and BMD (Figure 4(h)) of trabecular bones, PCA treatment at the dose of 20 mg/kg was better than E_2_ treatment. These results indicate that PCA might be preventive against OVX-mediated deterioration of trabecular bone architecture.

### 3.4. Effects of PCA on Serum RANKL, OPG, and RANKL/OPG Ratio in OVX Mice

**Figure 5:**
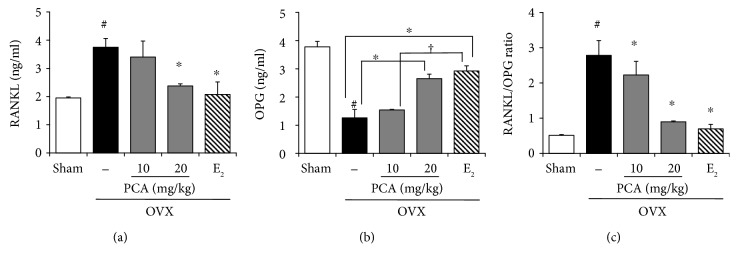
Effects of PCA on serum levels of RANKL and OPG and RANKL/OPG ratio. OVX mice received vehicle, PCA (10 and 20 mg/kg b.w./day), or E_2_ for 12 weeks. At the end of the experiment, sera were obtained; RANKL (a) and OPG (b) levels were determined using commercial ELISA kits; and the RANKL/OPG ratio was calculated (c). The results are expressed as mean ± SD (each group, *n* = 6). ^#^*p* < 0.05 between the sham and OVX-alone group. ^∗^*p* < 0.05 among OVX groups with or without interventions. ^†^*p* < 0.05 among OVX groups with interventions. b.w.: body weight; E_2_: 17*β*-estradiol; OPG: osteoprotegerin; OVX: ovariectomized; PCA: protocatechuic acid; RANKL: receptor activator of nuclear factor *κ*B ligand.

Excessive bone resorption would be ameliorated by inhibiting the activity of osteoclasts and osteoclast differentiation. Similar to E_2_, PCA supplementation effectively attenuated OVX-mediated trabecular bone destruction (Figure 4). Bone resorption is controlled by regulatory factors involving the tumor necrosis factor (TNF)/TNF receptor families, RANK, RANKL, and OPG [14, 15]. In addition, the balance between OPG and RANKL produced by osteoblasts is important for osteoclast regulation. The antiosteoporotic effect of PCA might be associated with its suppressive effect on bone resorption. To test this hypothesis, we examined changes in serum levels of OPG and RANKL, important regulators of the bone resorption process, in OVX mice in the presence or absence of PCA (each group, *n* = 6). The serum level of RANKL was significantly increased in OVX mice (Figure 5(a)), whereas the OPG level was significantly decreased (Figure 5(b)). As a result, the RANKL/OPG ratio was significantly higher than that of the sham controls (sham versus OVX group, 0.51 ± 0.02 versus 2.78 ± 0.41, *p* < 0.05). These results indicate that deterioration of trabecular bones observed in OVX mice is, at least in part, associated with enhancement of bone resorption. Like E_2_, PCA treatment at the dose of 20 mg/kg not only significantly decreased the serum level of RANKL but also increased the OPG level. Thereby, the RANKL/OPG ratio was significantly decreased compared to that of OVX mice (Figure 5(c)).

### 3.5. Effects of PCA on the Expression of TRAF6 and NFATc1 in OVX Mice

**Figure 6:**
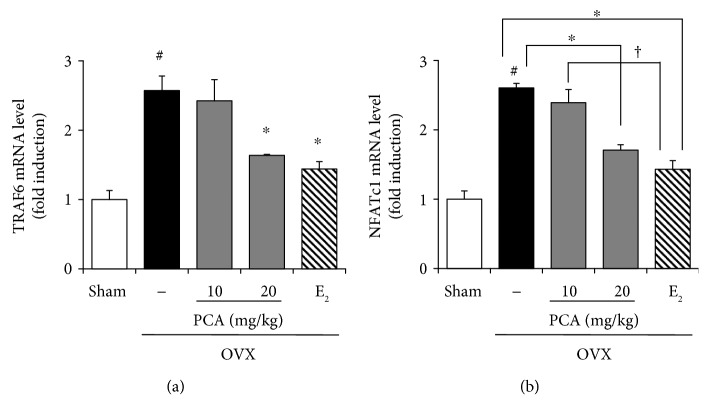
Effects of PCA on mRNA expression level of TRAF6 and NFATc1. OVX mice received vehicle, PCA (10 and 20 mg/kg b.w./day), or E_2_ for 12 weeks. At the end of the experiment, bone marrow cells were isolated, and the mRNA expression levels of cathepsin K (a) and calcitonin receptor (b) were measured by real-time RT-PCR. *β*-Actin was used as a loading control for RT-PCR. The results are expressed as a mean ± SD (each group, *n* = 6). ^#^*p* < 0.05 between the sham and OVX-alone group. ^∗^*p* < 0.05 among OVX groups with or without interventions. ^†^*p* < 0.05 among OVX groups with interventions. b.w.: body weight; E_2_: 17*β*-estradiol; OVX; ovariectomized; PCA: protocatechuic acid; TRAF6: TNF receptor-associated factor-6; NFATc1: nuclear factor of activated T cell cytoplasmic 1.

During osteoclast differentiation, binding of RANKL to its receptor RANK results in recruitment of TRAF6, leading to activation of downstream signaling molecules such as NFATc1 [15]. As shown in Figure 5(a), PCA supplementation (20 mg/kg) significantly reduced the serum level of RANKL in OVX mice. We therefore assumed that the expression level of TRAF6 and NFATc1 would be repressed in the presence of PCA. Indeed, both TRAF6 and NFATc1 mRNA levels in bone marrow cells of PCA- (20 mg/kg) supplemented OVX mice were significantly lower than those in OVX mice (*p* < 0.05, Figure 6). Collectively, these data show that PCA supplement suppressed the signaling pathways involved in osteoclast differentiation and thereby reduced the impairment of trabecular bone architecture in OVX mice (Figure 4).

### 3.6. Effects of PCA on Expression of Cathepsin K and Calcitonin Receptor in OVX Mice

**Figure 7:**
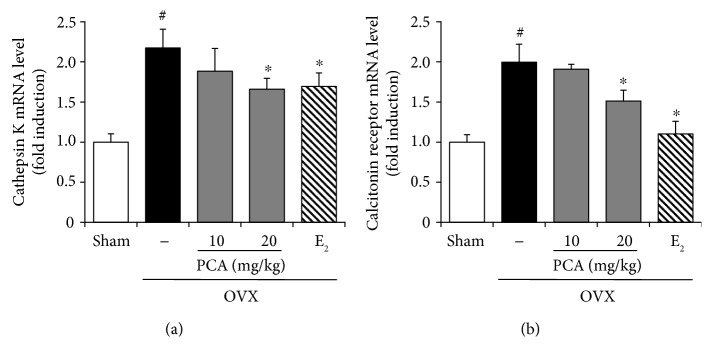
Effects of PCA on the mRNA expression level of cathepsin K and calcitonin receptor. OVX mice received vehicle, PCA (10 and 20 mg/kg b.w./day), or E_2_ for 12 weeks. At the end of the experiment, bone marrow cells were isolated, and mRNA expression levels of cathepsin K (a) and calcitonin receptor (b) were measured by real-time RT-PCR. *β*-Actin was used as a loading control for RT-PCR. The results are expressed as mean ± SD (each group, *n* = 6). ^#^*p* < 0.05 between the sham and OVX-alone group. ^∗^*p* < 0.05 among OVX groups with or without interventions. b.w.: body weight; E_2_: 17*β*-estradiol; OVX; ovariectomized; PCA: protocatechuic acid.

To further test the suppressive effects of PCA on osteoclast differentiation, the expression levels of cathepsin K and calcitonin receptor, osteoclast-specific markers in the bone marrow cells, were determined in OVX mice (each group, *n* = 6). The OVX mice showed an approximately 2-fold increase in the mRNA expression levels of cathepsin K and calcitonin receptor compared to the sham controls (*p* < 0.05). However, supplementation with PCA at the dose of 20 mg/kg or E_2_ significantly suppressed the increase in both cathepsin K and calcitonin receptor (Figure 7).

### 3.7. Effects of PCA on Serum Levels of Osteocalcin and BALP in OVX Mice

**Figure 8:**
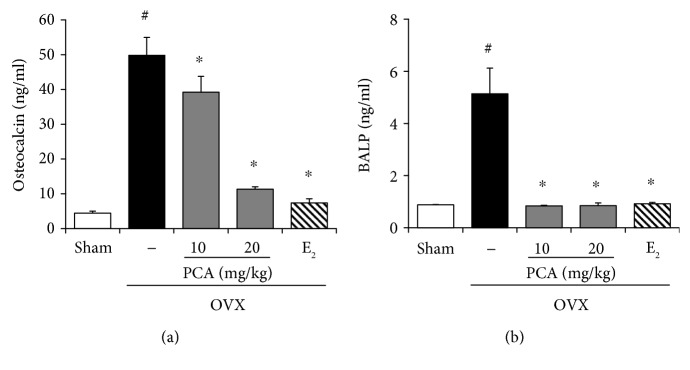
Effects of PCA on serum levels of osteocalcin and BALP. OVX mice received vehicle, PCA (10 and 20 mg/kg b.w./day), or E_2_ for 12 weeks. At the end of experiment, sera were obtained, and osteocalcin (a) and BALP (b) levels were determined using commercial ELISA kits. The results are expressed as mean ± SD (each group, *n* = 6). ^#^*p* < 0.05 between the sham and OVX-alone group. ^∗^*p* < 0.05 among OVX groups with or without interventions. BALP: bone alkaline phosphatase; b.w.: body weight; E_2_: 17*β*-estradiol; OVX; ovariectomized; PCA: protocatechuic acid.

Osteocalcin and BALP, which are synthesized by osteoblasts, are released into the circulation during the bone resorption process [10]. Increased levels of osteocalcin and BALP in serum indirectly reflect progression toward bone resorption. As shown in Figure 8, the levels of both osteocalcin and BALP were significantly increased in the OVX group, but these effects were significantly suppressed in the presence of PCA (20 mg/kg) and E_2_ (*p* < 0.05). The inhibitory potential of 20 mg/kg PCA against BALP was comparable to that of E_2_. These results suggest that PCA supplementation can suppress bone resorption initiated by osteoblasts in OVX mice.

## 4. Discussion

In this study, oral administration of PCA to OVX mice prevented loss of the tibial bone, preserved trabecular bone microarchitecture, and improved bone biomechanical properties. In conjunction with this result, administration of PCA (20 mg/kg body weight) normalized serum levels of RANKL, OPG, and osteocalcin in OVX mice. The mRNA expression levels of TRAF6 and NFATc1, which are involved in the RANKL-RANK signaling pathway, were significantly suppressed by PCA administration. In addition, the mRNA expression levels of calcitonin receptor and cathepsin K were also significantly suppressed by PCA. Thus, PCA reduced the bone resorption caused by estrogen deficiency through suppression of signaling pathways involved in the activation of osteoclasts.

It has been suggested that the degeneration of the uterus observed in OVX mice represents a model for the bone loss due to estrogen deficiency that occurs in women after menopause [29]. Ovariectomy results in a significant decrease in uterine weight, BMD of trabecular bone, and biomechanical strength, in part due to estrogen deficiency. In our study, the body weight of mice was increased in the OVX group (Figure 1(a)), which is consistent with previous reports [30]. The uterus is one of the most estrogen-responsive reproductive tissues and predominantly expresses estrogen receptor (ER) *α* [31]. In contrast to the increase in body weight, the OVX mice showed initial atrophy of the uterus that was recovered by treatment with E_2_ (Figure 1(b)). Considering that supplementation with PCA had no significant effect on the uterus but could ameliorate the impaired density and architecture of trabecular bone with similar potential to E_2_ supplementation (Figure 4), it is assumed that PCA has less estrogenic activity or suppresses loss of bone in a different manner.

In the evaluation of bone structure, BV/TV and BMD are known to be critical parameters for determining the fragility of trabecular bone [8]. Thus, a lower value of BV/TV not only indicates fewer trabecular bones but also is associated with morphological features such as rod-shaped and disconnected trabecular bones [32]. PCA administration resulted in increased BV/TV (bone volume ratio), consistent with the improvement in BMD (Tb). In addition, PCA supplementation restored the trabecular connectivity by increasing Tb.N (trabecular number) and reducing Tb.Sp (trabecular spacing). PCA increased both BS/BV (bone surface to volume) and Tb.Th (trabecular thickness) compared to the OVX group (Figure 4).

Osteoclasts are specialized cells involved in degradation of bone matrix. Among more than 24 involved genes or loci, osteoclast differentiation and activation are largely regulated by the action of OPG, RANK, and RANKL [33, 34]. OPG and RANKL are produced by osteoblastic cells, with a balance between membrane-bound RANKL and secreted OPG decoy receptor. The RANK signaling pathway is negatively regulated by OPG [35]. The differentiation of osteoclast cell precursors is induced upon the binding of RANKL and RANK, which promotes the activation of mature osteoclasts. Therefore, RANKL is an essential factor for differentiation, activation, and survival of osteoclasts in bone remodeling, whereas OPG is a soluble decoy receptor and inhibitor of RANKL action. It seems that loss of trabecular bone induced by OVX or estrogen insufficiency might be associated with signaling pathways involved in the acceleration of bone resorption. From this view of point, supplementation of PCA normalized the RANKL/OPG ratio, which is a good marker of severe osteolysis, by increasing the production of OPG and downregulating the production of RANKL (Figure 5). Alternatively, increased levels of osteocalcin and BALP, which are synthesized by osteoblasts and considered markers of bone turnover, were significantly attenuated by treatment with PCA or E_2_ compared to the OVX control group (Figure 8). It seems that the administration of PCA might also affect osteoblast activity, although the underlying mechanisms require further study.

TRAF6 has been shown to be a major adaptor molecule in signal transduction of RANK-RANKL, leading to activation of NFATc1 [36, 37], which functions as a major regulator of osteoclastogenesis via upregulation of osteoclast-specific genes such as cathepsin K, calcitonin receptor, osteoclast-associated receptor (OSCAR), and *β*3 integrin, in concert with transcription factors such as activator protein-1 (AP-1) , PU.1, microphthalmia-associated transcription factor [36]. In the present study, the mRNA expression levels of TRAF6 and NFATc1 were significantly increased in OVX mice, but these increases were suppressed by administration of PCA (Figure 6). The expression of other signaling molecules involved in osteoclast differentiation, such as cathepsin K and calcitonin receptor, in OVX mice was significantly inhibited by supplementation with PCA (Figure 7).

**Figure 9:**
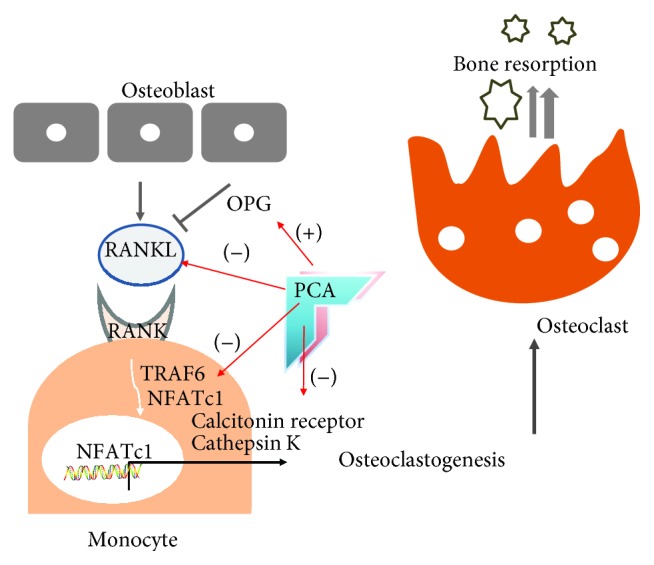
Summary of inhibitory potential of PCA against osteoclastogenesis in OVX mice. NFATc1: nuclear factor of activated T cell cytoplasmic 1; OPG: osteoprotegerin; PCA: protocatechuic acid; RANK: receptor activator of nuclear factor *κ*B; RANKL: RANK ligand; TRAF6: TNF receptor-associated factor-6; (+): increase; (-): decrease.

In summary, the inhibitory potential of PCA against osteoclastogenesis, which augments bone resorption in OVX or postmenopausal conditions, was demonstrated in the OVX mouse model. As summarized in Figure 9, the underlying mechanism of PCA in the suppression of bone loss in OVX mice may be associated with the following effects: (1) reduction of serum level of RANKL and increase in OPG; (2) blocking the RANK signaling pathway via downregulation of TRAF6 and NFATc1 expression; and (3) attenuation of cathepsin K and calcitonin receptor expression. PCA shows promise as a starting compound or alternative to estrogenic constituents in the development of antiosteoporotic compounds with improved safety. The exact signaling target of PCA involved in the suppression of osteoclastogenesis and/or improved bone formation mediated by osteoblasts in OVX mice remains unclear. This issue should be further investigated in future research to develop new antiosteoporotic compounds based on the action of PCA.

## Figures and Tables

**Table 1:** Primer sequences used for real-time PCR.

Gene name	Primer sequences
Cathepsin K	5′-CACCCAGTGGGAGCTATGGAA-3′ (forward)
	5′-GCCTCCAGGTTATGGGCAGA-3′ (reverse)

Calcitonin receptor	5′-AGGCAGACCCAAATGCTGTAATG-3′ (forward)
	5′-TTGGTGATAGGTTCTTGGTGACCTC-3′ (reverse)

TRAF6 (tumor necrosis factor receptor-associated factor-6)	5′-TTAAATGTCGGCATTCTCAGGGTA-3′ (forward)
	5′-TTGTGACCGAGACTCTCCCAAG-3′ (reverse)

NFATc1 (nuclear factor of activated T cell cytoplasmic 1)	5′-GCTTCACCCATTTGCTCCAG-3′ (forward)
	5′-ATGGTGTGGAAATACGGTTGGTC-3′ (reverse)

*β*-Actin	5′-TCACCCACACTGTGCCCATCTACGA-3′ (forward)
	5′-GGATGCCACAGGATTCCATACCCA-3′ (reverse)

**Table 2:** Effects of PCA on weight of the thymus and spleen in OVX mice.

Tissue (mg)		OVX			
	Sham	—	PCA (mg/kg b.w.)		E_2_
			10	20	(0.1 mg/kg b.w.)
Thymus	47.75 ± 2.06	61.66 ± 5.68^#^	61.33 ± 18.50	36.0 ± 1.41^∗^	44.0 ± 2.45^∗^
Spleen	151.65 ± 0.71	94.42 ± 3.79^#^	88.08 ± 2.65	117.11 ± 1.41^∗^	137.14 ± 8.47^∗^

Data are mean ± SD (each group, *n* = 6). ^#^*p* < 0.05 between the sham and OVX-alone group. ^∗^*p* < 0.05 among OVX groups with or without interventions. b.w.: body weight; E_2_: 17*β*-estradiol; OVX: ovariectomized; PCA: protocatechuic acid.

## Data Availability

The data used to support the findings of this study are available from the corresponding author upon request.

## Abbreviations

BALP: Bone alkaline phosphatase
BMD: Bone mineral density
CT: Computed tomography
E2: 17β-estradiol
OPG: Osteoprotegerin
NFATc1: Nuclear factor of activated T cell cytoplasmic 1
OVX: Ovariectomized
PBS: Phosphate-buffered saline
PCA: Protocatechuic acid
RANK: Receptor activator of nuclear factor kappa B
RANKL: Receptor activator of nuclear factor *κ*B ligand
RT-PCR: Reverse transcription polymerase chain reaction
TARP: Tartrate-resistant acid phosphatase
TRAF6: Tumor necrosis factor receptor-associated factor-6
b.w.: Body weight
i.p.: Intraperitoneal.
